# Getting old in the desired gender: a systematic review on aging diseases in transgender people

**DOI:** 10.1007/s40618-024-02353-y

**Published:** 2024-06-21

**Authors:** C. Ceolin, M. V. Papa, A. Scala, G. Sergi, A. Garolla

**Affiliations:** 1 Geriatrics Division, Department of Medicine (DIMED), University of Padua, via Giustiniani 2, 35128 Padua, Italy; 2 Regional Reference Center for Gender Incongruence (CRRIG), Padua, Veneto Italy; 3 Department of Neurobiology, Care Sciences and Society, Karolinska Institutet and Stockholm University, Aging Research Center, Stockholm, Sweden; 4 Unit of Andrology and Reproductive Medicine, Department of Medicine (DIMED), University of Padua, Padua, Italy

**Keywords:** Transgender, Aging, Older adults, Gender incongruence

## Abstract

**Introduction:**

The growing demographic presence of the transgender (TGD) population has sparked an increase in clinical investigations focusing on the impacts of gender-affirming hormone therapy (GAHT) in adults with gender dysphoria. Despite this surge in studies, there remains a significant gap in the literature regarding the health status of older TGD individuals. This review aims to assess prevalent pathological conditions within the TGD population, specifically concentrating on aging-related diseases investigated to date.

**Methods:**

A systematic search across Embase Ovid, Scopus, PubMed, Cochrane Library, and Web of Science databases was conducted to identify articles reporting on the aging process in TGD individuals. Methodological quality was evaluated using Newcastle–Ottawa Scale (NOS) scores.

**Results:**

Initial database searches yielded 12,688 studies, which were refined to 18 through elimination of duplicates and title/abstract review. Following a comprehensive appraisal, nine studies were included in the systematic review. These articles, published between 2017 and 2023, involved a total of 5403 participants. The evidence indicates a noteworthy percentage of the TGD population being at risk for cardiovascular diseases, experiencing depression or disability, and demonstrating hesitancy toward major recommended screening programs.

**Conclusions:**

Limited studies on older TGD individuals highlight not only an organic risk of chronic diseases but also a cognitive/psychiatric risk that should not be underestimated. Further research is imperative to deepen our understanding of the pathophysiological mechanisms involved in the health challenges faced by older TGD individuals.

**Supplementary Information:**

The online version contains supplementary material available at 10.1007/s40618-024-02353-y.

## Introduction

Transgender is an umbrella term encompassing individuals whose gender identity, whether transient or persistent, differs from the sex assigned at birth. Transgender and Gender Diverse (TGD) individuals may identify as male or female, or embrace a non-binary identity. Those whose gender identity aligns with the sex assigned at birth are referred to as cisgender [1]. In scientific literature, the terms "assigned female at birth" (AFAB) and "assigned male at birth" (AMAB) are frequently utilized in the context of TGD individuals. It's worth noting that these terms are also applicable to cisgender individuals, indicating the sex assigned at birth [2]. The terms 'trans women' and 'trans men' are also widely utilized. Gender dysphoria (GD) is a condition marked by psychological distress associated with gender incongruence [3]. Effective gender-affirming care for TGD individuals necessitates multidisciplinary management, including mental health care, gender-affirming hormone therapy (GAHT), and/or gender-affirming surgery (GAS) [1, 4]. GAHT often involves testosterone for transgender AFAB (t-AFAB) individuals and a combination of estrogen and anti-androgen drugs (e.g., cyproterone acetate, spironolactone) for transgender AMAB (t-AMAB) individuals [1, 4].

Current estimates suggest that approximately 0.6% of the population identifies as TGD [5]. Specifically, the prevalence of GD requiring medical intervention, such as hormone therapy or gender affirmation surgery, among adults (> 18 years) is estimated to be around 0.005–0.014% for individuals assigned male at birth and 0.002–0.003% for those assigned females at birth [5]. In Italy, there are approximately 300,000 TGD individuals, with a prevalence of 1.2–2.7% among adolescents, demonstrating an upward trend [1, 6].

Given these demographic trends and the increasing life expectancy, healthcare providers worldwide should address the unique needs of 'geriatric' TGD individuals to ensure successful aging. The aging process has been extensively studied in cisgender individuals, defined as a dynamic, irreversible, and complex physiological process occurring in the biological, psychological, and social spheres [7]. Distinctive aging events, such as mitochondrial dysfunction, telomere attrition, epigenetic alterations, genomic instability, imbalanced metabolism, and stem cell depletion, interact to increase susceptibility to chronic diseases [8]. Although the aging process is highly variable and subjective, gender differences have been observed, with women exhibiting greater longevity and men displaying greater strength, higher physical performance test scores, and, on average, less frailty compared to women [8]. These differences may be attributed to varying chromosomal patterns and the influence of sex hormones throughout the lifespan. Sex hormones play a pivotal role in the prominent endocrine changes linked to aging, exhibiting distinct effects in males and females not only during puberty but also in the early stages of aging [8]. Consequently, the progression of menopause and andropause differs, as women undergo a more significant decline in sex hormones compared to men, who frequently remain asymptomatic probably due to a progressive and slower decline in testosterone levels. A shorter reproductive lifespan in women is associated with decreased longevity and an increased risk of cardiovascular events but a lower risk of mortality from gynecological tumors. In men, higher testosterone levels are linked to a lower cardiovascular risk and mortality from cancer [8].

Despite the extensive number of studies (exceeding 10,000 from 2017 to 2023) published on the health of TGD individuals in recent years, the lack of prospective studies focusing on TGD individuals over 45 years poses significant clinical implications and represents a crucial research gap that needs addressing [9]. Consequently, we conducted this systematic review to identify and critically appraise studies investigating the aging process in TGD individuals. We highlight the deficiencies in current research and propose new research avenues, particularly in the context of the effects of feminizing and masculinizing therapies during aging.

## Methods

### Systematic review tool

This review adheres to PRISMA (http://www.prisma-statement.org/) and Meta-Analysis of Observational Studies in Epidemiology (MOOSE) guidelines [10].

### Study identification

The Cochrane Library, Embase Ovid, PubMed, and Web of Science databases were systematically queried for articles containing the terms 'transgender,' 'aged,' and 'gender nonconforming' up to October 2023. The detailed search string utilized for bibliographic retrieval is provided in the Supplementary Materials. Only papers and reviews in the English language were considered. The focus of interest encompassed studies involving TGD individuals aged 45 years or older. Additionally, references cited in the selected papers underwent examination to identify any potentially relevant articles. The initial article selection, conducted by the primary reviewer M.V.P., underwent a comprehensive validation process. This involved the repetition and confirmation of the entire process by a second reviewer (C.C.) to ensure the accuracy and validity of the inclusion criteria. In cases where differences of opinion arose, a thorough discussion ensued until consensus was reached on the inclusion or exclusion of a study, facilitated by a third reviewer (A.G.).

### Risk of bias assessment

Quality assessment was conducted independently by 2 reviewers (M.V.P. and C.C.) following Newcastle–Ottawa Scale (NOS) criteria for observational studies.

### Selection criteria

Inclusion criteria were: (1) articles on TGD people age ≥ 45 years; and (2) articles dealing with any health conditions linked to the aging process. Exclusion criteria were: (1) case reports, abstracts, letters, and editorials; (2) studies not written in English.

### Data extraction

The titles and abstracts of the selected articles were screened for relevance, and the following data were extracted from them: (1) study design; (2) sample size; (3) median/mean age of participants; (4) relevant findings.

## Results

**Fig. 1:**
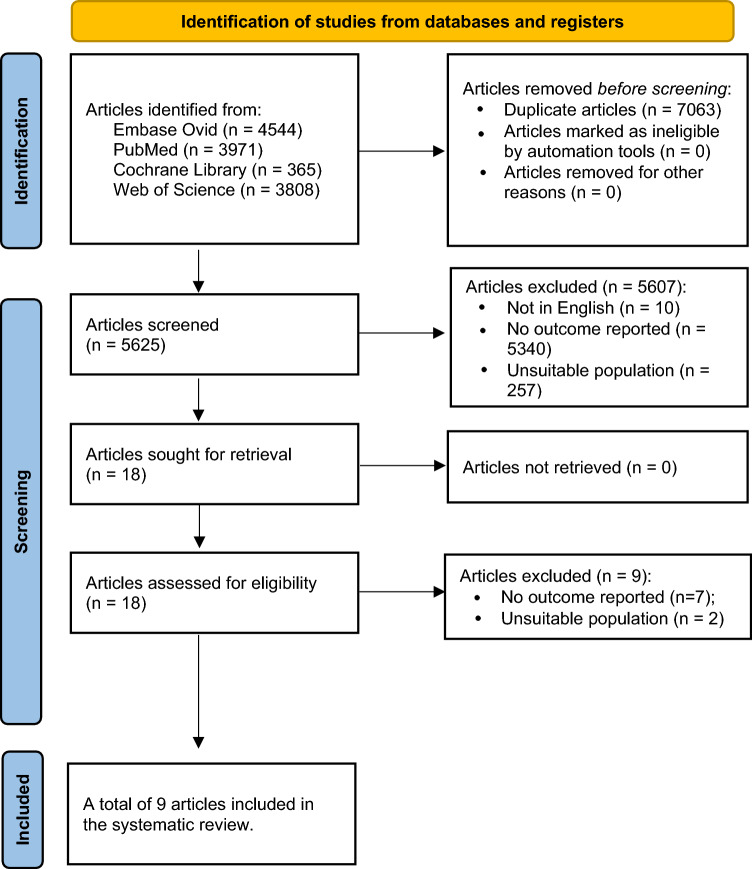
PRISMA flow diagram of the study selection process with reasons for inclusion and exclusion

A total of 12,688 studies were initially identified through the database searches, with 7063 duplicates subsequently excluded. Upon the review of titles and abstracts, 5607 studies were eliminated as they did not adhere to the inclusion criteria. Out of the remaining 18, an additional nine were excluded due to factors such as inappropriate populations or missing data, resulting in a final selection of nine papers. The full manuscripts of these nine papers were then thoroughly assessed for eligibility, as illustrated in Fig. 1. The quality assessments for these nine studies are extensively detailed in Table 1. Following evaluation by two researchers, eight studies were categorized as 'good,' while one study was labeled as 'fair.' The average Newcastle–Ottawa Scale score was determined to be 7.4, indicating an overall high level of quality in the selected studies.

**Table 1:** Study quality assessment using the Newcastle–Ottawa Scale (NOS) for observational studies

Author/year	Selection				Comparability (matched analysis)	Outcomes			NOS score
	Consecutive or obviously representative series of cases	Representativeness of exposed cohort	Ascertainment of exposure	Demonstration that outcome of interest was not present at the start of study		Assessment of outcome	Follow up long enough for the outcome	Adequacy of follow-up of cohorts	
Hoy-Ellis et al. 2022	*	*	*	*	*	*	*	*	8
Fredriksen-Goldsen et al. 2017	*	*	*	*	-	*	*	*	7
Jung et al. 2023	*	*	*	*	*	*	-	-	6
Alzahrani et al. 2019	*	*	*	*	*	*	*	*	8
Kiran et al. 2019	*	*	*	*	*	*	*	-	7
Li et al. 2023	*	*	*	*	*	*	*	*	8
Balcerek et al. 2021	*	*	*	*	*	*	*	*	8
Getahun et al. 2018	*	*	*	*	*	*	*	*	8
Poteat et al. 2021	*	*	*	-	*	*	*	*	7

*NOS* Newcastle–Ottawa quality assessment scale

*Represent fulfillment of the individual criterion within the subsection

### Study characteristics

Out of the nine papers included in the review, two were observational studies, two were retrospective studies, and five were cross-sectional studies. All of these studies specifically focused on the aging processes within TGD populations, meeting the previously mentioned inclusion criteria. The selected studies encompassed a total of 5403 participants and were conducted in the USA [12–19] and Canada [19]. The publication dates of these studies ranged from 2017 to 2023.

Table 2 provides a comprehensive overview of all the selected studies, outlining the primary results concerning the aging processes observed in TGD individuals.

**Table 2:** Selected studies investigating aging processes in TGD people and their main findings

Author/year/reference	Study design	Country	Sample size	Mean/median age	Findings
Fredriksen-Goldsen et al. (2017) [11]	Cross-sectional study	USA	411	61.41 ± 0.24	The most common pathological conditions in LGBT people were depressive symptomatology 32.05%, poor general health 26.08%, chronic health conditions 3.10%, disability 53.12%.
Hoy-Ellis et al. (2022) [12]	Cross-sectional study	USA	161	61.0 ± 8.1	Regarding preventive health screenings, older transgender people were significantly less likely to undertake physical examination (OR: 0.58, p < 0.01), colonoscopy (OR: 0.43, p < 0.01), mammogram (OR: 0.08, p < 0.01), pap smear (OR: 0.06, p < 0.01), osteoporosis test (OR: 0.08, p < 0.01), and PSA test (OR: 0.22, p < 0.01) than cisgender participants.
Jung et al. (2023) [13]	Cross-sectional study	USA	197	61.3	The most common pathological conditions in transgender people were depressive symptomatology 37.5%, lifetime discrimination and victimization 6.6%, and disability 47.3%.
Alzahrani et al. (2019) [14]	Retrospective cohort study	USA	3055	53.1	The transgender population had a higher reported history of myocardial infarction compared with the cisgender population, except for transgender women compared with cisgender men, even after adjusting for cardiovascular risk factors.
Kiran et al. (2019) [19]	Cross-sectional study	Canada	38	> 50	Transgender people were less likely to undergo recommended screening for cervical, breast, and colorectal cancer compared with cisgender people. Adjusted ORs comparing the likelihood of trans vs cisgender individuals being screened for cancer were 0.46 for uterine cervix cancer, 0.28 breast cancer, 0.51 colorectal cancer.
Li et al. (2023) [15]	Observational study	USA	88	> 50	Transgender older adults deal with social marginalisation and stigmatisation by generating an "authenticated social capital", a form of social capital built by developing alternative social networks and support systems that affirm their identity and foster authenticity and wellbeing.
Balcerek et al. (2021) [16]	Cross-sectional study	USA	55	52.8	The most prevalent CVD risk factors in transgender people were: hypertension (29%), current smoker (24%), obesity (20%), dyslipidaemia (16%), and diabetes (9%).
Getahun et al. (2018) [17]	Retrospective cohort study	USA	1380	> 45	In the transgender population, individuals on feminizing therapy have a greater incidence of cardiovascular mortality than individuals on masculinizing therapy. Transfeminine participants had a higher incidence of venous thromboembolism, with 2- and 8-year risk differences of 4.1 and 16.7 per 1000 persons, respectively, relative to cisgender men, and 3.4 and 13.7, respectively, relative to cisgender women.
Poteat et al. (2021) [18]	Observational study	USA	114	53.5 (51.12–55.97)	No meaningful differences between cisgender and transgender participants were found in smoking habits or CVD conditions. However, there were increased odds of VTE among transgender women compared to cisgender women. The rate of any CVD condition in the trans population was 38.5%. Adjusted OR of any CVD condition trans vs. cis was 0.79.

*LGBT* lesbian, gay, bisexual, and transgender, *CVD* cardiovascular

### Aging diseases

Fredriksen-Goldsen et al. conducted a study in the USA, enrolling 411 TGD individuals aged 50 years and older, with a mean age of 61.41 ± 0.24 ﻿[11]. TGD older adults reported elevated rates of job-related discrimination throughout their lives. Notably, over half (51.40%) of t-AMAB identified individuals reported not being hired for a job due to their perceived identity. Approximately 29% of TGD individuals were identified as "midlife bloomers," individuals who became aware of and disclosed their LGBT identity in their mid-40s, primarily comprising retired individuals. Another classification included 21.40% of individuals, both t-AFAB and t-AMAB, categorized as "beleaguered at-risk", signifying those who recognized their LGBT identity in adolescence and first disclosed it in their mid-20s. Additionally, "retired survivors" and "visibly resourced" accounted for 12.1% and 12.30% of TGD individuals, respectively. These classifications represented individuals who became aware of their LGBT identity during adolescence, experiencing significantly high rates of work-related discrimination throughout their lives (retired survivors), and individuals who became aware of their LGBT identity in late adolescence, disclosing it around the age of 25 (visibly resourced) ﻿[11]. These were the most prevalent clusters identified by the authors. Noteworthy, there were no significant differences between the clusters regarding health outcomes and quality of life, although the "visibly resourced" group exhibited lower depressive symptomatology and lower levels of perceived stress. Approximately 53.12% of the entire sample reported having a disability, defined as limitations in physical activity due to physical, mental, or emotional problems, or requiring special equipment. Higher rates of disability were observed in "midlife bloomers" and "beleaguered at-risk" (62.61% and 61.10%, respectively), while cognitive impairment was more frequent in the "beleaguered at-risk" group (25.22%) ﻿[11].

In alignment with this study, Jung and colleagues reported disability and depressive symptomatology rates of 47.3% and 37.5%, respectively, in a sample of 2450 LGBT individuals, including 197 TGD individuals ﻿[13]. Disability was defined broadly, encompassing serious difficulties in seeing, hearing, walking, or climbing stairs, difficulties in concentration, memory, or decision-making, and difficulties in dressing, bathing, or running errands alone. Approximately 39% of the sample demonstrated "healthy behaviors and minimal barriers," indicating average probabilities of health-risk behaviors and high probabilities of health-promoting behaviors. Another 31% were classified as having "less healthy behaviors and high barriers," with higher-than-average probabilities of risk-taking behaviors, lower probabilities of health-promoting behaviors, and limited use of preventive care ﻿[13]. The remaining categories included "healthy behaviors and healthcare system barriers" (19%) and "optimal health behaviors with risks of limited access to healthcare" (11%). The former exhibited higher probabilities of healthcare system barriers, while the latter demonstrated minimal probabilities of health-risk behaviors, high probabilities of health-promoting behaviors, low probabilities of using preventive screening, and moderate probabilities of healthcare barriers. Detailed percentages of TGD individuals within each class were not reported ﻿[13].

### Cardiovascular risk

In a survey involving 3055 TGD individuals (1267 t-AFAB and 1788 t-AMAB), with a mean age of 53 years, a higher incidence of myocardial infarction was observed in t-AFAB individuals compared to cisgender women (7.2% vs. 3.1%, p < 0.01) ﻿[14]. Conversely, t-AMAB individuals exhibited significantly higher rates of myocardial infarction compared to both cisgender women (7.8% vs. 3.1%, p < 0.01) and cisgender men (7.8% vs. 5.6%, p < 0.01). Moreover, TGD individuals were more frequently current smokers and less physically active than their cisgender counterparts ﻿[14]. After correcting for factors such as diabetes mellitus, smoking habits, chronic kidney disease, exercise, and age, it was found that t-AMAB individuals have a higher risk of myocardial infarction only when compared to cisgender women. Meanwhile, t-AFAB individuals were four times more likely to experience a myocardial infarction compared with cisgender women and twice as likely compared with cisgender men ﻿[14].

Balcerek et al. focused on cardiovascular risk in 296 TGD individuals stratified into two age groups (< 45 and ≥ 45 years) ﻿[16]. The total estradiol dose was similar between groups, with the ≥ 45-years group primarily receiving transdermal estradiol at a higher average dose than those treated orally (6 mg vs. 4 mg, p = 0.01). The prevalent cardiometabolic risk factors in the ≥ 45-years group included hypertension (29%), current smoking (24%), obesity (20%), dyslipidemia (16%), and diabetes (9%). Rates of ischemic heart disease, cerebrovascular disease, and venous thromboembolic disease were less than 5% ﻿[16].

Getahun and colleagues investigated adverse cardiovascular events in 1,380 TGD individuals (1,044 t-AMAB and 336 t-AFAB) aged 45 and over, recording 61 and 23 venous thromboembolisms, 54 and 16 ischemic strokes, and 33 and 9 myocardial infarctions in the t-AMAB and t-AFAB populations, respectively ﻿[17]. In t-AMAB individuals, a longer follow-up period with estrogen therapy correlated with worse cardiovascular outcomes, but there was insufficient evidence to draw conclusions about the risk in t-AFAB individuals ﻿[17].

In a comparative study involving 114 TGD individuals (mean age 53.5 years) and 964 cisgender individuals, Poteat et al. found no meaningful differences in smoking habits or cardiovascular conditions between the two groups ﻿[18]. The odds of reporting any cardiovascular disease conditions significantly increased with heightened psychological distress in both TGD and cisgender participants. However, TGD individuals demonstrated increased odds of venous thromboembolism compared to cisgender counterparts, with t-AFAB individuals exhibiting an odds ratio of 3.35 (95% confidence interval: 1.07–10.46) compared to cisgender women. In t-AMAB individuals, the risk was higher only when compared to cisgender women (odds ratio: 3.94, 95% confidence interval: 1.24–12.51) but not compared to cisgender men ﻿[18].

### Screening tests and disease prevention

Hoy-Ellis et al. conducted a cross-sectional study comparing 161 TGD individuals with a mean age of 65 years to 2349 cisgender LGBT individuals to assess screening service adherence [12]. The TGD population exhibited significantly lower uptake of physical examinations and various screening tests, including colonoscopy, mammogram, pap smear, and osteoporosis and prostate-specific antigen (PSA) tests [12]. After adjusting for age, income, education, and race/ethnicity, TGD participants showed significantly lower odds of adhering to four out of the eight recommended screenings: mammogram [odds ratio (OR): 0.08, p < 0.01], pap smear (OR: 0.05, p < 0.01), osteoporosis test (OR: 0.08, p < 0.01), and PSA test (OR: 0.31, p < 0.01). t-AFAB individuals were notably less likely to have undergone a stool test or a colonoscopy (OR: 0.27 and OR: 0.39, respectively, p < 0.05) [12].

In a similar vein, Kiran and colleagues reported comparable results in a study comparing 38 TGD individuals aged 50 years and older with cisgender counterparts [19]. Crude uptake rates of screening tests among the transgender population were significantly lower than in the cisgender population for cervical cancer (56% vs. 72%, p = 0.001), breast cancer (33% vs. 65%, p < 0.001), and colorectal cancer (55% vs. 70%, p = 0.046). After adjusting for age and the number of visits, TGD patients were significantly less likely to adhere to screening for cervical cancer (OR: 0.39, 95% CI 0.25–0.62), breast cancer (OR: 0.27, 95% CI: 0.12–0.59), and colorectal cancer (OR: 0.50, 95% CI: 0.26–0.99) [19].

### Relationships during aging

A study delved into the life-course processes through which TGD older adults develop alternative social networks and support systems that validate their identities [15]. The investigation included 88 TGD individuals aged 50 years and above, who were queried about their life courses, with a specific emphasis on the role of relationships in either impeding or facilitating their gender expression [15]. In response to social marginalization and stigmatization, TGD older adults construct what is termed "authenticated social capital"—a type of social capital crafted by establishing alternative social networks and support systems that validate their identity and promote authenticity and well-being [15]. The authors underscore that the conventional forms of social capital that TGD individuals possess through connections with their family and community of origin prove detrimental to their ability to lead an authentic life. Consequently, trans older adults forge alternative forms of social capital as a means to counteract inequality and social oppression [15].

## Discussion

We conducted a comprehensive systematic review of studies focusing on aging in TGD individuals. The findings indicate a significant risk of cardiovascular disease, along with occurrences of depression and disability within the TGD population. Additionally, there is a noticeable reluctance to adhere to key recommended screening programs. Nevertheless, there remains a notable gap in available data concerning the aging process in TGD individuals, leaving numerous questions unanswered.

Undoubtedly, the aging trajectory of the TGD population is shaped by social determinants of health at both individual and community levels. Importantly, there persists a deficiency in social support and the provision of inclusive, dignified care for TGD individuals [20]. Specifically, the prevalence of homophobia, transphobia, rejection, and discrimination experienced by TGD individuals, collectively termed "minority stress," disproportionately impacts their overall health and carries significant clinical implications [20]. For instance, individuals enduring minority stress face an elevated risk of developing depression and other psychiatric conditions [21]. Furthermore, apprehension about potential judgment from healthcare professionals leads to a reluctance among TGD individuals to seek medical attention, whether for primary care or screening procedures [21]. Lastly, while limited data are currently available, minority stress could potentially contribute to the observed higher mortality rates in the TGD population [22].

The subsequent sections of this review expound upon the primary areas addressed and the associated evidence.

### The real risk of cognitive impairment

TGD individuals appear to face a heightened risk of cognitive impairment and Alzheimer's disease (AD) compared to their cisgender counterparts [9]. Elevated levels of stress hormones, linked to accelerated brain aging and cognitive decline, alongside multiple stressors, may contribute to negative self-perceptions and maladaptive psychological phenomena, triggering physical and mental health disorders [23]. Furthermore, increased cortisol levels, dysregulation of the hypothalamic–pituitary–adrenal axis, and the cumulative physiological toll of prolonged stress, known as "allostatic load," can lead to functional and structural damage throughout the body [23]. Considering these factors, it is plausible that minority stress plays a significant role in the onset of dementia [23]. Moreover, there are notable disparities in the prevalence of various modifiable risk factors for both dementia and AD among TGD individuals. Key data highlight significantly higher rates of depression and smoking in TGD individuals (both t-AFAB and t-AMAB), along with higher rates of diabetes and obesity [24]. Previous studies have indicated a considerably higher prevalence of depression in TGD women compared to the cisgender population, with a lifetime prevalence of 62% versus 16.6% for the general US population [25]. This is concerning given that depression is a well-established risk factor for AD in later life [24]. Additionally, some studies have reported that TGD individuals are nearly twice as likely to experience mild cognitive impairment (MCI) and more than twice as likely to report related functional limitations, such as reduced ability to work, volunteer, or socialize, compared with women or men [26].

Cognitive aging in the TGD population has received limited attention thus far, necessitating further studies to gain a better understanding of potential mechanisms, including the specific impact of minority stress on cognition in LGBT individuals.

### Behind the cardiovascular risk

While the constituents of feminizing therapy have evolved over time, emerging evidence suggests that TGD individuals may face a heightened cardiovascular risk compared to their cisgender counterparts. Regrettably, studies focusing on individuals aged 50 and above are notably scarce. Proposed pathophysiological mechanisms include increased body fat and insulin resistance, alongside compromised endothelial function in t-AMAB individuals [27]. Additionally, platelet activation leading to elevated coagulation and inflammation markers has been implicated [27]. T-AFAB individuals appear to exhibit elevated lipids, including total cholesterol, triglycerides, and low-density lipoprotein, as well as increased levels of high-sensitivity C-reactive protein [27]. Conversely, testosterone therapy may elevate hematocrit, potentially impacting vascular health. While the incidence of vascular complications due to secondary erythrocytosis from masculinizing GAHT is reassuringly low, further research is imperative to comprehensively comprehend the clinical implications of erythrocytosis in this context [28]. Lastly, the escalation of cardiovascular risk factors in transgender individuals, encompassing smoking habits, inadequate nutrition, and sedentary lifestyles, assumes a pivotal role; minority stress associated with gender dysphoria is posited as a major catalyst for these unhealthy lifestyle habits.

### Cancer risk

In the course of the gender affirmation process, TGD individuals are subjected to prolonged and high-dose administration of exogenous hormones [29]. Consequently, the potential carcinogenic effects of GAHT in aging TGD individuals have become a subject of notable interest and concern. However, comprehensive investigations into the impact of long-term masculinizing and feminizing GAHT on cancer risk in older TGD individuals remain limited, primarily due to the relatively youthful age of participants in existing studies. The absence of well-defined guidelines further compounds the challenge in assessing cancer risk among TGD individuals undergoing masculinization or feminization therapy [28]. Studies conducted in younger TGD individuals indicate several factors implicated in neoplastic risk, including a higher prevalence of sexually transmitted infections, increased exposure to recognized risk factors such as smoking and alcohol use, and insufficient access to adequate screening [30, 31]. Reported cases of malignancies linked to hormones in transgender women undergoing gender affirmation treatments encompass breast and prostate carcinomas, prolactinomas, and meningiomas [32, 33]. For transgender men, clinical reports describe tumors in the breast, ovaries, cervix, vagina, and endometrium [29]. Despite the reported overall incidence of 0.04% for prostate cancer in t-AFAB individuals, the actual incidence of prostate and breast cancer in the TGD population remains uncertain due to limitations in existing cohort studies. Some studies even suggest that the incidence may be lower or at least comparable to that among cisgender individuals [29, 34–36]. However, with advancing age, the risk increases, and the influence of environmental factors and lifestyle should not be underestimated.

Surveys conducted in Europe between 2013 and 2014 highlight that 46–80% of TGD individuals are sexually active, with a majority being heterosexual, favoring anal sex, and engaging in multiple sexual partnerships [28]. Additionally, sexually active t-AMAB individuals display a lower inclination to use condoms during receptive anal sex with their primary partners. Financial challenges sometimes propel individuals towards engaging in high-risk sexual behaviors, including having multiple sex partners, unprotected sex, and involvement in commercial sex work [28]. Consequently, TGD individuals face a substantial risk of HIV infection and other sexually transmitted infections, such as human papillomavirus (HPV), potentially leading to a higher risk of cervical, anal, and oropharyngeal cancers. However, the actual prevalence of cervical high-risk HPV infection in the overall transmasculine adult population remains unknown [28].

### Unresolved questions for future research

A notable deficiency in current research pertains to the scarcity of data on bone health in TGD individuals. Studies focusing on young TGD adults reveal diminished bone mineral density (BMD) values in t-AMAB individuals undergoing feminizing therapy even before the initiation of GAHT [37, 38]. In comparison to their cisgender counterparts, reduced BMD is associated with higher cortical porosity, lower trabecular density and number, and increased trabecular separation [38, 39].  The underlying reasons remain unclear, but lifestyle factors may exert a significant influence, with low engagement in outdoor physical activities, suboptimal nutritional habits, and particularly smoking possibly compromising the attainment of peak bone mass [37, 38]. Studies conducted during GAHT have suggested that, after approximately 12–24 months, masculinizing hormone therapy in t-AFAB individuals is not significantly linked to changes in Bone Mineral Density (BMD). Conversely, feminizing hormone therapy in t-AMAB individuals has been associated with increased BMD at the lumbar spine [40, 41]. Unfortunately, limited data are available on the risk of fractures in TGD patients. Wiepjes et al. examined fractures, obtained from emergency department data, in TGD patients in comparison to the cisgender population [41]. Fracture risk was higher in older t-AMAB individuals only when compared to age-matched cisgender men (OR: 1.90, 95% CI: 1.32–2.74) but was lower in t-AFAB individuals compared to cisgender men (OR: 0.57, 95% CI: 0.35–0.94) [41]. Additionally, AMAB individuals were reported to have lower grip strength and reduced levels of total and appendicular lean mass [37]. The long-term implications of these findings, such as potential complications in muscle performance across various body systems or areas, remain unknown.

Recently, a new taxonomic entity known as "couplepause" has been emerging. This term refers to the consequences of hormonal and age-related changes that can lead to an alteration in sexual functionality within a couple, thereby concretizing the combined impact of menopause and andropause on the sexual health of older couples, resulting in simultaneous age-related changes for both members of the couple [42]. This underscores the importance of adopting an approach that is no longer centered solely on the individual but rather directed towards the couple, to comprehensively address the sexual health needs of elderly couples [42, 43]. Challenges associated with aging in couple dynamics primarily involve hormonal decline, coupled with relational issues, changes in social strength, and concurrent illnesses [43]. Recently, a group of experts has proposed enhancing the education of couples, utilizing specific educational materials to facilitate understanding of age-related changes. This actively involves both men and women in collaboratively managing symptoms and self-care. Furthermore, the working group emphasizes the importance of providing comprehensive training for healthcare professionals so that they can acquire the necessary skills to address sexual health during consultations [43]. Despite the term "couplepause" having been predominantly used so far to describe the impacts of menopause and andropause on heterosexual couples, attention should also be directed towards the sexual health of TGD individuals. Aspects such as gender transition, hormone therapies, and related surgeries may be significant elements influencing couple sexual health, especially with aging. The current lack of literature on this topic highlights the need to delve into the theme of "couplepause" in these individuals as well.

### Limitations

One primary limitation of the existing research is its predominant focus on higher-income populations, thereby lacking representativeness for the global population. Few studies have been conducted in low- and middle-income countries (LMIC), potentially restricting the generalizability of the findings. Moreover, certain studies contain possible confounders that might influence the ultimate results. We acknowledge that certain strategies, such as the inclusion of studies only in the English language, could have enhanced the specificity of our literary search. Furthermore, we extended our research to include individuals aged 45 or older, which does not align with the WHO guidelines for the elderly category. However, this decision allowed us to select a greater number of articles in light of the limited literature available on older individuals. Nevertheless, we did not impose restrictions on the publication timeframe of studies. Additionally, we conducted a thorough analysis of citations from key articles to identify further related research, and we scrutinized the bibliographies of relevant articles and literature reviews to uncover additional pertinent sources. We believe these considerations contribute valuable strengths to our work. Furthermore, our review's strength lies in its specific emphasis on transgender individuals aged 45 and above. Given the scarcity of evidence regarding the life expectancy of transgender individuals, our study stands out as one of the few investigations concentrating on older populations experiencing gender dysphoria.

## Conclusion

With the rising number of TGD individuals, there is a growing focus on the long-term impacts of GAHT. The limited existing studies on older TGD individuals indicate that, beyond the inherent risks of chronic diseases, there is a noteworthy cognitive and psychiatric risk that should not be overlooked. Additional research is imperative to elucidate the underlying pathophysiological mechanisms. This, in turn, will enhance awareness among healthcare professionals and contribute to ensuring the successful aging of individuals experiencing gender incongruence.

### Supplementary Information

Below is the link to the electronic supplementary material.

Supplementary file1 (DOCX 12 KB)

## Abbreviations

TGD: Transgender and gender diverse
AFAB: Assigned female at birth
AMAB: Assigned male at birth
t-AFAB: Transgender assigned female at birth
t-AMAB: Transgender assigned male at birth
GD: Gender dysphoria
GAHT: Gender-affirming hormone therapy
GAS: Gender-affirming surgery
NOS: Newcastle–Ottawa Scale

## Declarations

### Data availability

All data generated or analyzed during this study are included in this published article.
