# Supplementary material for: Getting old in the desired gender: a systematic review on aging diseases in transgender people
Source: J Endocrinol Invest. 2024 Jun 21;47(8):1851–62. doi: 10.1007/s40618-024-02353-y (PMC11266207; doi:10.1007/s40618-024-02353-y)
Supplement: Supplementary file 1 — Supplementary file1 (DOCX 12 KB) [file 40618_2024_2353_MOESM1_ESM.docx]

(transgender OR “Person, Transgender” OR “Two-Spirit Persons” OR Transexual* OR “gender nonconforming” OR "non-binary" OR genderqueer OR queerness OR queer OR "gender dysphoria”) AND (aged OR elderly OR old OR aging OR geriatric OR “advanced age” OR senior).
